# Inhalation

**Published:** 1875-05

**Authors:** E. H. Gibbs


### INHALATION.

BY E. H. GIBBS, A.M., M.D.

THE treatment of diseases of the throat and lungs by inhalation of medicated vapors, is no doubt very effective when properly administered; that is, when the process employed is such as to bring the remedies in contact with the diseased parts. The apparatus usually employed for this purpose consists of a crucible in which the ingredients are placed, from which the gas or vapor is generated by heat. Above the crucible is the receiver where the vapor is confined. To this receiver is attached a flexible tube through which the vapor passes into the mouth or nostrils of the patient. For the treatment of local affections of the nares and mouth, where it may be deemed advisable to use pungent remedies, the form of "Inhaler" above described would certainly accomplish the object. But for the treatment of diseases of the throat and lungs, it is practically worthless, because the vapor cannot reach the parts without producing strangulation. It is not surprising, therefore, that the testimony of many who have been treated for diseases of the throat and lungs in this way, is not favorable to medicinal inhalation, and for the simple reason that they were inhaling only atmospheric air.

The only reliable method of accomplishing the object desired, is to impregnate thoroughly with the vapor of the medicines, the air which the patient is compelled to breathe. A small room should be fitted up for this purpose, and kept thoroughly ventilated. There should be nothing in the room, such as poisonous wall paper, soiled carpet or old cushioned chairs, upon which the vapor could act and form deleterious compounds which might be fatal to the occupant. The most scrupulous attention should be paid to these details in order to ensure the best results.

The method of generating the vapor is very simple. It may be done by mixing the medicines in the proper proportions and vaporising with a spirit lamp. This answers very well for temporary use. But where establishments are expressly fitted up for the treatment of patients, every valuable improvement should be adopted. It is desirable to have the medicated vapor thoroughly and evenly mixed with the pure air of the vapor room. Several instruments have been invented for this purpose. Those we have seen are all good. In our own practice we use "Outwater's Vaporizer," and think well of it.

We have described the process of applying vapor in affections of the throat and lungs. But its use is not confined to these. In skin diseases and some forms of rheumatism, the favorable effects of hot vapor of sulphur, applied to the entire surface of the body is often astonishing.

---

Three friends has a man in his life—wealth, family and good deeds. In the hour of his death, wealth and family avail not; but his good deeds have gone before him to make his pathway peace.

---

Hug the stove all winter, and thus sap the foundations of your constitution.

